# Thidiazuron-induced direct organogenesis from immature inflorescence of three date palm cultivars

**DOI:** 10.1186/s43141-021-00115-4

**Published:** 2021-01-20

**Authors:** Rania A. Taha, Mai A. Allam, S. A. M. Hassan, Basem M. M. Bakr, Mona M. Hassan

**Affiliations:** 1 Tissue Culture Technique Lab., Pomology Department, Agriculture and Biology Research Division and Central Laboratories Network, National Research Centre (NRC), 33 Elbohouth St., Dokki, Giza, 12622 Egypt; 2 Plant Biotechnology Department, Genetic Engineering and Biotechnology Division, (Centre of Excellence for Advanced Sciences) National Research Centre, 33 Elbohouth St., Dokki, Giza, 12622 Egypt; 3 Academy of Scientific Research and Technology, 101 Kasr El-Ainy St, Cairo, 11694 Egypt; 4 Pomology Department, Agriculture and Biology Research Division, National Research Centre, Giza, Egypt; 5 The Central Laboratory of Date Palm Researches and Development, Agriculture Research Center, Giza, Egypt; 6 Regional Development Centre of New Valley, Academy of Scientific Research and Technology, 101 Kasr El-Ainy St, Cairo, 11694 Egypt

**Keywords:** Inflorescence, Organogenesis, *Phoenix dactylifera*, Thidiazuron

## Abstract

**Background:**

Inflorescence explants of date palm proved to be a promising tool for micropropagation of elite cultivars or rare males and females as organogenesis and somatic embryogenesis could be achieved. These plant materials are abundantly available every year and can be used as cheap and potent explants. Nevertheless, many difficulties could be faced in this protocol according to selection of the spathe size and age, media components, growth regulators, etc. The aim of this study was to determine the influence of various cytokinins on direct organs induction of three date palm cultivars (Selmi, Barhee, and Medjool) from immature inflorescence. An additional objective of this study was to investigate the effect of cytokinins and auxins on growth and development of Medjool cultivar.

**Results:**

Various combinations of cytokinins were investigated on three date palm inflorescences as N6-(2-isopentenyl) adenine (2iP), kinetin, benzyleadenine (BA), and thidiazuron (*N*-phenyl-*N*′-1,2,3-thidiazol-5-yl urea) (TDZ). TDZ alone or in combination with BA proved to be superior for direct organogenesis in all three cultivars so that another combination of TDZ with BA was conducted. Results showed that moderate concentration of BA, with TDZ, gave superior response. Medjool cultivar response surpassed other two cultivars that made the possibility to conduct some growth regulators treatments on its multiplication and regeneration. TDZ at 0.5 + BA at 1.0 mg/l without activated charcoal seemed to enhance multiplication rate. Medium containing 0.5 mg/l of both naphthaleneacetic acid and indole butyric acid in addition to 1.0 mg/l indole acetic acid appeared to be more suitable for rooting stage of Medjool shootlets.

**Conclusion:**

In this study, we created an innovation sequence of growth regulators included in nutrient media for date palm direct organogenesis from inflorescence. Organogenesis has been accelerated from immature inflorescence explants and developed to healthy plantlets which acclimatized in greenhouse.

## Background

Date palm (*Phoenix dactylifera* L.) is a dioecious, perennial monocot plant species that belongs to family Arecaceae. It is one of the oldest fruit crops spreadly cultivated in North Africa and Middle East countries and one of the most economically important plants in arid and hot regions. Offshoots are traditionally used for vegetative propagation of date palms. However, tissue culture is the most used technology method to provide large-scale propagation of healthy true-to-type plants. For decades, shoot tip explants have been used for various micropropagation protocols of date palm on the research and commercial levels together [1]. Somatic embryos could be formed on date palm explants in an indirect way as it begins with callus induction [2, 3] or in a direct way as it begins firstly with bud differentiation [4]. For decades, date palm micropropagation has been performed with induction of indirect somatic embryogenesis. This technique with indirect organogenesis are currently used in many laboratories in the world to micropropagate date palm [5]. Although long time is required for the initiation phase, moderate multiplication rate is achieved and the strong influence of the variety is limiting the rate of micropropagation [6]

Recently, the inflorescence explants proved to be promising tool for micropropagation of elite cultivars and rare male and female individuals of date palm [7] as organogenesis and somatic embryogenesis could be achieved. These plant materials are abundantly available every year and can be used as they are cheap and potent. Nevertheless, many problems have been faced to achieve this technique: obtaining the explants at the right time and preferable size, determining the suitable composition of the starting medium and all other stages, and selecting the type and concentrations of various growth regulators needed for this technique. Inflorescence explants showed different types of responses during the date palm initiation stage due to the composition of the starting medium: direct vegetative buds [8], direct embryogenic cells that maturated into direct somatic embryos or shoots [9, 10], or unfriable callus [11].

Organogenesis is a technique which usually consists of four steps: initiation of vegetative buds, bud multiplication, shoot elongation, and rooting. Its success is highly dependent on the success of the first step [12]. Although this technique could produce true-to-type plants but provide low plant numbers.

In fact, the cytokinin type and concentration are very important factors that should be studied for organogenesis technique. Thidiazuron and benzyladenine are the most preferably used cytokinins in the regeneration systems in some fruit trees and woody plants [13]. Thidiazuron or TDZ (*N*-phenyl-*N*′-1,2,3-thidiazol-5-yl urea) has recently emerged as a highly efficient bioregulator of plant morphogenesis in the tissue culture technique. Application of TDZ induces diverse responses ranging from induction of callus to embryogenesis or organogenesis. TDZ is believed to act as an auxin in some cases and as a cytokinin in others. Nevertheless, it has a different structure from either auxins or purine-based cytokinins. TDZ can influence some physiological and biochemical reactions in plant cells. Various reports indicated that TDZ may act through modulation of the endogenous plant growth regulators, modification in cell membranes, energy levels, nutrient uptake, or nutrient assimilation [14].

The aim of this current study was to determine the role of various cytokinins especially thidiazuron on induction of direct shoot organs of three date palm cultivars (Selmi, Barhee, and Medjool) from immature inflorescence. An additional objective of this study was to investigate the effect of cytokinins and auxins on growth and regeneration of Medjool cultivar.

## Methods

The present study was carried out through 2018–2020 at the Tissue Culture Lab. of New Valley Regional Centre, Academy of Scientific Research and Technology, Egypt.

### Plant material and in vitro organogenesis establishment

**Fig. 1:**
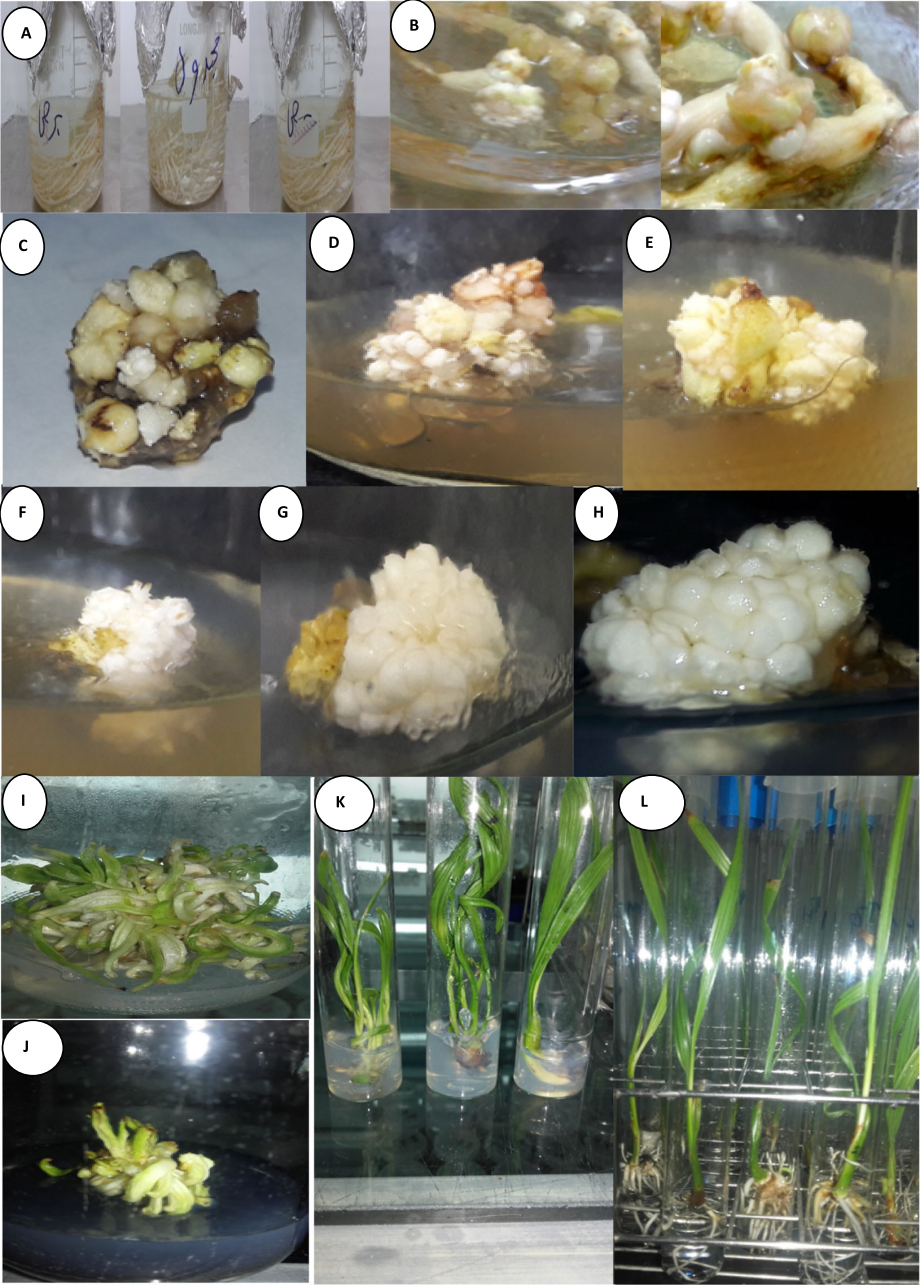
In vitro regeneration via direct organogenesis from immature inflorescence explants of date palm cultivars. **a** Sterilization of date palm inflorescences. **b** Swilling of immature inflorescence buds. **c** Organogenesis induction of Selmi explants cultured on medium containing 2.0 mg/l TDZ. **d** Organogenesis induction of Barhee explants cultured on medium containing 2.0 mg/l TDZ. **e** Organogenesis induction of Medjool explants cultured on medium containing 1.0 BA + 1.0 TDZ. **f** Organogenesis numbers of Selmi explants cultured on medium containing 2.0 mg/l TDZ + 1.0 mg/l BA. **g** Organogenesis numbers of Barhee explants cultured on medium containing 2.0 mg/l TDZ + 1.0 mg/l BA. **h** Organogenesis numbers of Medjool explants cultured on medium containing 2.0 mg/l TDZ + 1.0 mg/l BA. **i** Medjool shoot formation on medium containing 0.5 mg /l TDZ+ 1.0 mg /l BA. **j** Medjool shoot formation on medium containing 0.5 mg/l TDZ + 1.0 mg/l BA + AC. **k** Medjool shoot elongation. **l** Medjool root formation on medium containing (mg/l) 1.0 IAA + 0.5 NAA + 0.5 IBA

Immature female inflorescences of date palm (*Phoenix dactylifera* L.) cvs. Medjool, Barhee, and Selmi were removed from adult female trees grown at a respected farm in Cairo-Alexandria road, Egypt. Immature inflorescence spathes were collected in late February 2018. The average spathe length was 10–15 cm. Spathes were rinsed under running tap water and liquid soup for half an hour. Surface sterilization in laminar air flow is performed by using 20% of commercial Clorox (5.25% sodium hypochlorite) for 5 min and then rinsed with sterilized distilled water three times. After that, the spathes’ protective sheath was removed, and the spikelets were isolated and sterilized by immersion in 0.1% mercuric chloride (HgCl_2_) solution for 5 min (Fig. 1a). Sterilized spikelets were then rinsed with sterilized distilled water three times and divided into several segments each one contains 3–4 florets (explant).

Explants were cultured on a sterilized starting medium consisted of MS inorganic salts [15], 100 mg/l glutamine, 100 mg/l arginine, 2.0 g/l polyvinylpyrrolidone (PVP), 5.0 mg/l thiamine HCL, 1.0 mg/l biotin, 0.5 mg/l naphthaleneacetic acid (NAA), 1.0 mg/l naphthoxyacetic acid (NOA), 40 g/l sucrose, 100 mg/l inositol, 40 mg/l adenine sulfate and solidified with 6.0 g/l agar in addition to some types of cytokinins as thidiazuron (TDZ), benzyleadenine (BA), N6-(2-isopentenyl) adenine (2iP), or kinetin (Kin) with treatment concentrations as follows:

1. 2.0 mg/l 2iP
2. 2.0 mg/l Kin
3. 2.0 mg/l BA
4. 2.0 mg/l TDZ
5. 1.0 mg/l TDZ + 1.0 mg/l BA
6. 1.0 mg/l 2iP + 1.0 mg/l BA
7. 1.0 mg/l Kin + 1.0 mg/l BA

Cultures were incubated in darkness and recultured onto the same fresh medium every 6 weeks. After three subcultures, superior responses were achieved with the two treatments of 2.0 mg/l TDZ and 1.0 mg/ l TDZ + 1.0 mg/l BA, with the three cultivars under investigation. This observation led us to use some modifications of these media at the second experiment as follows:

1. 2.0 mg/l TDZ
2. 2.0 mg/l TDZ + 0.5 mg/l BA
3. 2.0 mg/l TDZ + 1.0 mg/l BA
4. 2.0 mg/l TDZ + 2.0 mg/l BA
5. 1.0 mg/l TDZ
6. 1.0 mg/l TDZ + 0.5 mg/l BA
7. 1.0 mg/l TDZ + 1.0 mg/l BA
8. 1.0 mg/l TDZ + 2.0 mg/l BA.

After each experiment, the explants were ranked into five classes based upon their browning intensity and swilling, from the lowest (1) to the highest (5) of the three cultivars. In addition, percentage of response (number of explants show bud formation/total cultured number × 100) and average number of organs appeared were recorded.

Due to higher percentage of the vegetative buds formation of Medjool cv., the following stages were done with this cultivar.

### Multiplication of vegetative buds of Medjool cv.

At this stage, clusters of vegetative buds (2–3 buds) were cultured on the following culture media which are dispensed on small jars (200 ml) at a rate of 35 ml/ jar to multiply and form new shoots. The culture media contained ¾ MS, 100 mg/l glutamine, 100 mg/l arginine, 5.0 mg/l thiamine HCL, 1.0 mg/l biotin, 80 mg/l adenine sulfate, 0.25 mg/l NAA, and 40 g/l sucrose solidified with 6.0 g/l agar. Cytokinins were added as follows: TDZ at 0.5 mg/l alone and combined with 0.5 mg/l BA, 1.0 mg/l BA, and 2.0 mg/l BA. Previous culture media were used with or without addition of 0.5 g/l activated charcoal. Cultured jars of Medjool cv. were maintained at 27 ± 2 °C and light intensity of 1500 lux, for three subcultures (1-month interval). After that, the number of new shootlets (buds) was calculated.

### Elongation stage

All shootlets from the previous treatments were transferred to ¾ MS + 0.5 mg/l kinetin + 1.0 mg/l indole acetic acid (IAA) for two subcultures (4-weeks interval). All cultures were incubated at 27 ± 2 °C and light intensity of 2000 lux in order to transfer to rooting media (Fig. 1k).

### Rooting stage

Vigorous shoots at lengths of 5–7 cm were transferred to half-strength MS media supplemented with 2.0 mg/l glycine, 170 mg/l NaH_2_PO_4_, 120 mg/l KH_2_PO_4_, and 40 g/l sucrose solidified with 6.0 g/l agar, in addition to the following auxin treatments: 1.0 mg/l IAA, 0.5 mg/l NAA, 0.5 mg/l indole butyric acid (IBA), or 0.5 mg/l NAA + 0.5 mg/l IBA. Culture media were dispensed into test tubes (2.5 in diameter and 25 cm in length) at a rate of 25 ml and capped with polyethylene closures. After 6 weeks, rooted plantlets were transferred to liquid culture media of the same components except sucrose which was reduced to 15 g/l and maintained 4 weeks as a pre-acclimatization treatment. Both solid and liquid culture media were incubated at light intensity of 4000 lux. Root number, root length, plantlet length, hairy roots, and root thickness degrees (estimated according to Pottino [16]) were recorded after solid media culture (6 weeks).

### Acclimatization stage

Healthy rooted plantlets were transferred to greenhouse. Acclimatization process was started by removing the plantlets from test tubes, rinsing them with tap water, immersing them in fungicide solution 0.2% (w/v) for 10 min and then transplanting them in plastic pots (5 cm in diameter and 18 cm in length) filled with moistened mixture of peat moss: perlite 2:1. The plantlets were covered with polyethylene bags for 6 weeks to raise the relative humidity around the plantlets. Bags were removed gradually, and plantlets were irrigated with 1/10 strength of MS inorganic medium.

### Statistical analysis

Each treatment of different experiments contained three replicates, and each replicate contained five jars and every jar cultured with five explants. Before analysis, percentage data were normalized by arcsine-transformation. Data obtained were subjected to the analysis of variances of randomized complete design as recommended by Snedecor and Cochran [17].

## Results

Immature inflorescence explants of three cultivars of date palm (Selmi, Barhee, and Medjool) were cultured in this investigation into media containing MS salts supplemented with 0.5 mg/l NAA, 1.0 mg/l NOA, 40 mg/l adenine sulfate, and 40 g/l sucrose. Various cytokinins were added to see their effect on direct organogenesis of the three cultivars.

### Effect of various cytokinins on browning and swilling degrees of three date palm explants after 4 months from initiation culture

Data from Table 1 revealed that Medjool showed the highest browning intensity compared with the other two cultivars. Medium containing 2.0 mg/l TDZ showed the highest browning intensity of the explants regardless of the cultivars’ factor, followed by TDZ combined with BA (1.0 mg/l for both). Similarly, it was found that Medjool showed the highest browning intensity with medium containing 2.0 mg/l TDZ followed by the combination of TDZ with BA (1.0 mg/l for both). Meanwhile, Selmi explants showed the lowest browning intensity with medium containing the combination of TDZ with BA.

**Table 1:** Effect of various cytokinins on browning intensity of three date palm cultivars after 4 months from initiation culture

Treatment (mg/l)	Barhee	Selmi	Medjool	Mean
2.0 BA	1.86^fg^	1.40 ^jk^	3.10^b^	2.12^B^
2.0 2iP	1.50^jk^	1.80^gh^	2.75^c^	2.02^C^
2.0 Kin	1.40^kl^	2.06^e^	2.80^c^	2.09^BC^
2.0 TDZ	1.70^hi^	1.60^ij^	3.88^a^	2.39^A^
1.0 BA + 1.0 TDZ	2.00^ef^	1.20^m^	3.20^b^	2.13^B^
1.0 BA + 1.0 2iP	1.64^ij^	2.25^d^	2.13^de^	2.01^C^
1.0 BA + 1.0 Kin	1.61^ij^	1.25^l^	3.20^b^	2.02^C^
Mean	1.67^B^	1.65^B^	3.01^A^	

Means with different letters were significantly different at 5% level

Furthermore, data in Table 2 and Fig. 1 (b) showed that Medjool explants gave the highest swelling degree compared with the other two cultivars, while Barhee explants showed the lowest swilling degree. Medium containing 2.0 mg/l TDZ showed the highest swelling degree of the explants regardless of the cultivars’ factor, followed by 2.0 mg/l 2iP while, combination of BA with kinetin (1.0 mg/l for both) showed the lowest swilling degree. It was found that Medjool explants showed the highest swelling degree with medium containing 2.0 mg/l TDZ followed by the combination of TDZ with BA. Meanwhile, Barhee explants showed the lowest swelling degree with medium containing the combination of 2iP with BA.

**Table 2:** Effect of various cytokinins on swilling degree of three date palm cultivars after 4 months from initiation culture

Treatment (mg/l)	Barhee	Selmi	Medjool	Mean
2.0 BA	1.43^jk^	1.54^j^	4.25^b^	2.41^D^
2.0 2iP	1.83^i^	2.30^h^	4.00^c^	2.71^B^
2.0 Kin	1.33^kl^	3.06^f^	3.25^e^	2.55^C^
2.0 TDZ	1.46^jk^	2.60^g^	5.00^a^	3.02^A^
1.0 BA + 1.0 TDZ	1.34^kl^	1.38^k^	5.00^a^	2.57^C^
1.0 BA + 1.0 2iP	1.11^m^	2.25^h^	3.75^d^	2.37^D^
1.0 BA + 1.0 Kin	1.20^l^	1.33^kl^	4.25^b^	2.26^E^
Mean	1.39^C^	2.07^B^	4.21^A^	

Means with different letters were significantly different at 5% level

### Effect of various cytokinins on response percentage and number of organs of three date palm explants after 4 months from initiation culture

Data in Table 3 and Fig. 1 (c, d, and f) indicated that Medjool showed the highest response percentage compared with the other two cultivars, followed by Barhee explants. Medium containing TDZ combined with BA (1.0 mg/l for both) showed the highest response percentage of the explants regardless of the cultivars’ factor, followed by TDZ alone at 2.0 mg/l. With respect to the interaction, it was found that Medjool showed the highest response percentage with medium containing the combination of TDZ with BA, followed by 2.0 mg/l TDZ. Meanwhile, Barhee explants showed response only with the medium containing TDZ alone (at 2.0 mg/l) or with combination with BA. In addition, Selmi explants showed response only with the medium containing TDZ alone (at 2.0 mg/l). Similarly, organ number had the same trend as response percentage as Medjool explants showed the highest results compared with the two other cultivars. In addition, medium containing TDZ showed higher results compared with 2iP and kinetin. With respect to the interaction, it was found that Medjool explants gave the highest organ number with medium containing the combination of TDZ with BA, followed by TDZ only at 2.0 mg/l (Table 4).

**Table 3:** Effect of various cytokinins on response percentage of three date palm cultivars after 4 months from initiation culture

Treatment (mg/l)	Barhee	Selmi	Medjool	Mean
2.0 BA	0.00^g^	0.00^g^	0.00^g^	0.00^F^
2.0 2iP	0.00^g^	0.00^g^	28.58^c^	9.53^C^
2.0 Kin	0.00^g^	0.00^g^	15.50^e^	5.17^D^
2.0 TDZ	19.0^d^	10.0^f^	30.40^b^	19.8^B^
1.0 BA + 1.0 TDZ	19.0^d^	0.00^g^	49.82^a^	22.94^A^
1.0 BA + 1.0 2iP	0.00^g^	0.00^g^	0.00^g^	0.00^F^
1.0 BA + 1.0 Kin	0.00 ^g^	0.00 ^g^	10.00^f^	3.34^E^
Mean	5.44^B^	1.44^C^	19.19^A^	

Means with different letters were significantly different at 5% level

**Table 4:** Effect of various cytokinins on organogenesis number of three date palm cultivars after 4 months from initiation culture

Treatment (mg/l)	Barhee	Selmi	Medjool	Mean
2.0 BA	0.00^g^	0.00^g^	0.00^g^	0.00^F^
2.0 2iP	0.00^g^	0.00^g^	8.77^c^	2.93^C^
2.0 Kin	0.00^g^	0.00^g^	7.57^d^	2.53^D^
2.0 TDZ	7.57^d^	6.89^e^	11.20^b^	8.55^A^
1.0 BA + 1.0 TDZ	8.68^c^	0.00^g^	13.07^a^	7.25^B^
1.0 BA + 1.0 2iP	0.00^g^	0.00^g^	0.00^g^	0.00^F^
1.0 BA + 1.0 Kin	0.00^g^	0.00^g^	5.00^f^	1.67^E^
Mean	2.33^B^	0.99^C^	6.52^A^	

Means with different letters were significantly different at 5% level

### Effect of various combinations of TDZ and BA on browning and swilling degrees of three date palm explants after 6 months from initiation culture

Data in Table 5 revealed that Medjool showed the highest browning intensity compared with the other two cultivars, followed by Barhee explants. Medium containing 2.0 mg/l TDZ showed the highest browning intensity of the explants regardless the cultivars’ factor, followed by TDZ combined with BA (2.0 and 0.5 mg/l, respectively). Similarly, it was found that Medjool showed the highest browning intensity with medium containing 2.0 mg/l TDZ followed insignificantly by the combination of TDZ with BA (2.0 and 0.5 mg/l, respectively). Meanwhile, Barhee and Selmi explants showed lower browning intensity in this stage.

**Table 5:** Effect of various combinations of TDZ and BA on browning intensity of three date palm cultivars after 6 months from initiation culture

Treatment (mg/l)	Barhee	Selmi	Medjool	Mean
1.0 TDZ	1.80^hijk^	1.77^ijkl^	2.20^de^	1.92^DE^
1.0 TDZ + 0.5 BA	1.75^jklm^	1.71^lmn^	2.19^de^	1.88^EF^
1.0 TDZ + 1.0 BA	1.71^lmn^	1.65^no^	2.17^e^	1.84^FG^
1.0 TDZ + 2.0 BA	1.68^mno^	1.60^o^	2.15^e^	1.81^G^
2.0 TDZ	2.00^f^	1.88^gh^	2.41^a^	2.10^A^
2.0 TDZ + 0.5 BA	1.89^g^	1.85^ghi^	2.35^ab^	2.03^B^
2.0 TDZ + 1.0 BA	1.89^g^	1.73^klmn^	2.31^bc^	1.98^C^
2.0 TDZ + 2.0 BA	1.83^ghij^	1.71^lmn^	2.26^cd^	1.93^CD^
Mean	1.82^B^	1.74^C^	2.25^A^	

Means with different letters were significantly different at 5% level

According to data in Table 6, results showed that Medjool explants gave the highest swelling degree compared with the other two cultivars, followed by Selmi explants. Medium containing 2.0 mg/l TDZ showed the highest swelling degree of the explants regardless the cultivars’ factor, followed by TDZ combined with BA (2.0 and 0.5 mg/l, respectively). Similarly, it was found that Medjool showed the highest swelling degree with medium containing 2.0 mg/l TDZ followed significantly by the combination of TDZ with BA (2.0 and 0.5 mg/l, respectively). Meanwhile, Barhee and Selmi explants showed lower swelling degrees with all treatments in this stage.

**Table 6:** Effect of various combinations of TDZ and BA on swilling degree of three date palm cultivars after 6 months from initiation culture

Treatment (mg/l)	Barhee	Selmi	Medjool	Mean
1.0 TDZ	1.96^k^	2.20^i^	2.76^d^	2.31^D^
1.0 TDZ + 0.5 BA	1.77^l^	2.07^j^	2.60^e^	2.15^E^
1.0 TDZ + 1.0 BA	1.79^l^	2.09^j^	2.57^e^	2.15^E^
1.0 TDZ + 2.0 BA	1.60^m^	1.90^k^	2.43^gh^	1.98^F^
2.0 TDZ	2.20^i^	2.50^f^	3.02^a^	2.57^A^
2.0 TDZ + 0.5 BA	2.17^i^	2.47^fg^	2.94^b^	2.53^B^
2.0 TDZ + 1.0 BA	2.09^j^	2.39^h^	2.89^bc^	2.46^C^
2.0 TDZ + 2.0 BA	2.08^j^	2.38^h^	2.85^c^	2.44^C^
Mean	1.96^C^	2.25^B^	2.76^A^	

Means with different letters were significantly different at 5% level

### Effect of various combinations of TDZ and BA on response percentage and number of organs of three date palm explants after 6 months from initiation culture

Data in Table 7 indicated that Medjool explants were the superior cultivars in response percentage followed by Barhee then Selmi explants. Incorporation of BA with TDZ was efficient for increasing the explant response percentage (number of responded explant/total number of cultured explants × 100). Data showed that medium containing 2.0 mg /l TDZ + 1.0 mg/l BA gave the highest response percentage, followed by medium containing 1.0 mg/l TDZ + 1.0 mg/l BA. Higher BA concentration (2.0 mg/l), when incorporated with TDZ, reduced the response percentage. With respect to the interaction, it was found that Medjool explants showed the highest response percentage with medium containing the combination of TDZ with BA (2.0 and 1.0 mg/l, respectively), followed by Medjool explants cultured on 1.0 mg/l TDZ + 1.0 mg/l BA and Barhee on 2.0 mg/l TDZ + 1.0 mg/l BA. The lowest response was shown on Selmi explants cultured on medium containing 1.0 mg/l TDZ.

**Table 7:** Effect of various combinations of TDZ and BA on response percentage of three date palm cultivars after six months from initiation culture

Treatment (mg/l)	Barhee	Selmi	Medjool	Mean
1.0 TDZ	9.63^l^	4.69^m^	15.33^j^	9.88^G^
1.0 TDZ + 0.5 BA	22.12^h^	16.37^ij^	28.00^g^	22.16^E^
1.0 TDZ + 1.0 BA	45.00^c^	38.21^e^	50.08^b^	44.43^B^
1.0 TDZ + 2.0 BA	17.29^i^	11.22^k^	22.73^h^	17.08^F^
2.0 TDZ	27.07^g^	22.16^h^	32.83^f^	27.36^D^
2.0 TDZ + 0.5 BA	40.03^d^	33.46^f^	44.08^c^	39.19^C^
2.0 TDZ + 1.0 BA	49.55^b^	40.18^d^	62.72^a^	50.82^A^
2.0 TDZ + 2.0 BA	22.40^h^	15.37^j^	28.21^g^	21.99^E^
Mean	29.14^B^	22.71^C^	35.50^A^	

Means with different letters were significantly different at 5% level

The same trend could be observed in Table 8 and Fig. 1 (f, g, and h) data as result showed that Medjool explants were the superior cultivars in number of organs appeared followed by Barhee and Selmi explants. Incorporation of BA with TDZ was efficient for increasing the number of organs. With respect to the interaction, it was found that Medjool explants showed the highest number of organs with medium containing the combination of TDZ with BA (2.0 and 1.0 mg/l, respectively), followed by Medjool explants cultured on 2.0 mg /l TDZ+ 0.5 mg/l BA and 1.0 mg /l TDZ+ 1.0 mg/l BA. Although Selmi and Barhee explants gave less organ number than Medjool explants, they gave the same trend as the highest number of organ could be achieved for these two cultivars was with medium containing 2.0 mg /l TDZ + 1.0 mg/l BA.

**Table 8:** Effect of various combinations of TDZ and BA on organogenesis number of three date palm cultivars after 6 months from initiation culture

Treatment (mg/l)	Barhee	Selmi	Medjool	Mean
1.0 TDZ	3.74^l^	2.55^m^	6.20^k^	4.16^F^
1.0 TDZ + 0.5 BA	6.05^k^	6.03^k^	11.08^c^	7.72^D^
1.0 TDZ + 1.0 BA	8.80^fgh^	8.83^fgh^	13.07^b^	10.23^B^
1.0 TDZ + 2.0 BA	4.24^l^	4.16^l^	9.18f^g^	5.86^E^
2.0 TDZ	7.47^j^	7.67^ij^	11.68^c^	8.94^C^
2.0 TDZ + 0.5 BA	8.43^ghi^	8.14^hij^	13.21^b^	9.93^B^
2.0 TDZ + 1.0 BA	9.44^ef^	10.17^de^	15.77^a^	11.80^A^
2.0 TDZ + 2.0 BA	5.99^k^	5.58^k^	10.25^d^	7.27^D^
Mean	6.77^B^	6.64^B^	11.30^A^	

Means with different letters were significantly different at 5% level

### Effect of benzyladenine concentrations and activated charcoal (AC) on multiplication of date palm cv. Medjool

Previous resulted organ cultures of Medjool cultivar were cultured on media containing 3/4 MS salts, 80 mg/l adenine sulfate, 0.25 mg/l NAA, 0.5 mg/l TDZ and various concentrations of BA to study the multiplication rate. Data in Table 9 reflected shoot number formation as affected by the previous mentioned treatments. Observations showed that the addition of BA to the culture media increased shoot number formation compared with culture medium without BA. The highest significant number was recorded with 1.0 mg/l BA. Increasing BA, from 1.0 to 2.0 mg/l, decreased significantly the shoot number. Moreover, no significant difference could be observed between 0.5 and 2.0 mg/l BA. Concerning activated charcoal addition, culture medium containing AC achieved lower shoot number compared with medium devoid of AC (Fig. 1i, j). Interaction in this respect revealed that increasing BA from 0 to 1.0 mg/l in the presence or absence of AC increased shoot number formation. The highest shoot number could be observed in medium supplemented with 1.0 mg/l BA without AC.

**Table 9:** Effect of benzyladenine with or without activated charcoal on new shoots formation of date palm cv. Medjool

Treatments (mg/l)	Activated charcoal	Free-activated charcoal	Mean
0.5 TDZ	18.38^cde^	16.89^e^	17.64^C^
0.5 TDZ + 0.5 BA	19.53^cd^	17.7^de^	18.52^BC^
0.5 TDZ + 1.0 BA	19.68^bc^	29.64^a^	24.66^A^
0.5 TDZ + 2.0BA	18.00^cde^	21.6^b^	19.8^B^
Mean	18.90^B^	21.46^A^	

Means with different letters were significantly different at 5% level

#### Rooting stage

Data in Table 10 shows the effect of different auxins on root system during rooting stage of date palm. The highest significant root number appeared with rooting medium supplemented with 0.5 mg/l of both NAA and IBA in addition to 1.0 mg/l IAA, followed by medium containing 0.5 mg/l IBA + 1.0 mg/l IAA. While, the lowest root number appeared at 1.0 mg/l IAA. In addition, rooting medium containing IAA alone surpassed other media in root length. It could be observed that the presence of NAA and IBA with IAA in rooting media achieved the highest significant degrees of root thickness, hairy root formation and plant length (Fig. 1 l) compared with IAA alone.

**Table 10:** Effect of auxin combinations on root formation of date palm cv. Medjool

Treatment (mg/l)	Root number	Root length	Root thickness	Hairy root degree	Plant length
1.0 IAA	2.2^c^	3.7^a^	1.67^b^	2.00^c^	8.90^a^
1.0 IAA + 0.5 NAA	3.1^b^	3.2^ab^	2.07^b^	2.57^c^	10.0^a^
1.0 IAA + 0.5 IBA	3.38^b^	2.77^b^	3.50^a^	3.6^b^	9.50^a^
1.0 IAA + 0.5 NAA + 0.5 IBA	4.25^a^	3.2^ab^	4.00^a^	4.83^a^	10.3^a^

Means with different letters within each column were significantly different at 5% level

## Discussion

Direct organogenesis presented in the current study was proliferated from immature inflorescence explants of three date palm cultivars (Barhee, Selmi, and Medjool). Immature inflorescence explants are a promising alternative source of explants as they provide a quick and safe method for micropropagation of date palms [18]. Direct organogenesis is usually used to produce clonal plants that are true-to-type, due to avoid of callus phase organization [12]. Histological studies of direct organogenesis revealed that adventitious buds are formed directly from epidermal cells without callus formation and they are developed from meristematic cells in the tissues [19]. Few researches have reported regeneration of date palm through organogenesis especially from inflorescence. Moreover, many factors like basal medium formulation, type and concentration of carbon source type, and concentration of plant growth regulators as well as plant genotype seem to be critical for date palm organogenesis [20]. Our study suggested that, at all levels, Medjool cultivar explants surpassed the two other cultivars in all data collected while Barhee and Selmi explants swapped locations with each other in some treatments. Similarly, Rad et al. [21] observed significant difference between Medjool and Mazafati cultivars as Medjool produced more vegetative buds than Mazafati. Date palm organogenesis was reported to be genotype-dependent [22].

TDZ proved to be crucial for organogenesis of the three cultivars under investigation, and this result is in harmony with Hassan et al. [23] as they found that the highest percentage of direct shoot buds and direct embryos formation occurred on MS supplemented with 1.0 mg l^−1^ TDZ combined with auxin. Moreover, benzyladenine incorporation in TDZ-medium enhanced explant response percentage and organ numbers. It was reported that the maximum response on medium supplemented with 1.0 mg/l BA and 0.5 mg/l TDZ was observed, producing an average of 18.2 buds per culture after 24 weeks from date palm culture [19]. TDZ at 2.0 mg/l also could achieve shoot sprouting directly on leaf tissues of *Urginea altissima* [24], at 1.5 mg/l for *Passiflora miniata* [25] and at 1.0 mg/l for *Aloe vera* [26] and *Arnebia euchroma* [27].

Many researchers tried to understand the role of action of TDZ in plant. For instance, Dey et al. [28] perceive that TDZ appears to stimulate cells in the apical meristem to divide and multiply then develop so that bud differentiation occurred. Mundhara and Rashid [29] believe that TDZ-ability to induce shoot bud production in the dark is triggered by calcium stress, which in turn affects the production of ethylene. The role of TDZ in morphogenesis is intimately related to the metabolism of endogenous growth regulators. Moreover, TDZ treatment increased levels of endogenous auxin, ethylene, and ABA [30, 31]. Interestingly, the applied concentration of 0.5 mg/l TDZ with 1.0 mg/l BA enhanced peroxidase activity during budding of date palm cv. Hillawi, where peroxidase activity was associated with increased number of buds formation [19].

Results show that incorporating BA with TDZ and other growth regulators in the media encourage proliferation as new shoots were accelerated in multiplication stage. The increase in shoot proliferation might be due to the physiological role of BA which is considered to be the most widely used cytokinin in the micropropagation industry due to its effectiveness and affordability [32]. It accelerates cell division and differentiation of adventitious buds. Histological sections revealed that additional shoot bud primordia were differentiated, due to BA presence, within the explants just underneath the supersized cells where their development is suppressed [33].

It could be observed from our results that the presence of NAA and IBA with IAA (0.5, 0.5, and 1.0 mg/l, respectively) in rooting media achieved the highest significant root number, root thickness, and hairy root formation on Medjool shootlets compared with IAA alone. The presence of NAA, in the rooting medium, increased root number of date palm somatic embryos. Either NAA or IBA at 0.4, 0.6 or 0.8 mg/l with 60 g/l sucrose gave the best root thickness. It was reported that the effect of NAA is on the main root length while IBA on the lateral root length [34]. Al-Mayahi [19] indicated that shoots were rooted on MS media supplemented with 0.2 mg/l of NAA. Rooted shoots were successfully acclimatized and established in a mixture of peat moss and perlite (2:1) with 80% success. Optimum rooting percentage 90% was achieved when shoots were transferred to a medium with 1.0 mg/L NAA. The average root number after 8 weeks was 5.4 with 9.0 cm length [35].

## Conclusions

In this study, we created innovation sequences of growth regulators included in nutrient medium for date palm direct organogenesis from inflorescences. Organogenesis has been accelerated from immature inflorescence explants of three cultivars. TDZ and BA are crucial cytokinins to be included into induction and multiplication media of date palm. Medjool proved to be a promising cultivar for micropropagation and biotechnology field, and its shoots were developed to healthy plantlets which acclimatized in greenhouse.

## Data Availability

The authors declared that all data are included in the article.

## Abbreviations

TDZ: Thidiazuron (*N*-phenyl-*N*′-1,2,3-thidiazol-5-yl urea)
NAA: Naphthaleneacetic acid
NOA: Naphtoxyacetic acid
BA: Benzyleadenine
2iP: N6-(2-Isopentenyl) adenine
Kin: Kinetin
HgCl_2_: Mercuric chloride
IAA: Indole acetic acid
IBA: Indole butyric acid
